# Development and evaluation of an automated classification and counting system for rice planthoppers captured on survey boards

**DOI:** 10.1038/s41598-025-05908-y

**Published:** 2025-07-01

**Authors:** Toshihisa Yashiro, Tomohiko Takayama, Ryo Sugiura, Masaya Matsumura, Sachiyo Sanada-Morimura

**Affiliations:** 1 Koshi Campus, Institute for Plant Protection, National Agriculture and Food Research Organization (NARO), Koshi, 861-1192 Japan; 2 Kurume Campus, Kyushu Okinawa Agricultural Research Center, National Agriculture and Food Research Organization (NARO), Kurume, 839-8503 Japan; 3 Research Center for Agricultural Information Technology, National Agriculture and Food Research Organization (NARO), Tsukuba, 305-0856 Japan

**Keywords:** Pest management, Artificial intelligence, You Only Look Once (YOLO), Agricultural pests, Rice pest insects, Ecology, Environmental sciences, Mathematics and computing

## Abstract

Rice planthoppers are the most economically important insect pests of rice in Asia. Traditional surveys to examine their abundance and composition in paddy fields involve human visual inspection, which requires considerable time and effort by expert entomologists. We previously developed a deep learning-based object detection system which can detect rice planthopper individuals from scanned images of survey boards, categorize, and count planthopper individuals by 18 categories, based on species, developmental stages, adult sexes, and adult wing-forms, with a mean average precision (mAP) of 79%. In this study, we modified the system by reconsidering the categories of planthopper individuals to be counted and by additional supervised training. The modified system can count rice planthopper individuals captured on survey boards across 17 categories with a mAP of 91%. We also showed that by using the system developed here, classification and counting of rice planthopper individuals can be completed in approximately six minutes per survey board, which can take more than an hour for human experts. Thus, this high-performance system can greatly save time and reduce labor costs for monitoring the occurrence, reproduction, and population growth of the rice planthoppers in paddy fields, which could increase the efficiency of their management.

**Supplementary Information:**

The online version contains supplementary material available at 10.1038/s41598-025-05908-y.

## Introduction

More than half of the world’s population depends on rice (*Oryza sativa* L.) as their staple crop^1^. In Asia, where more than 90% of the world’s rice is produced, rice planthoppers, *Nilaparvata lugens* (Stål) (brown planthopper), *Sogatella furcifera* (Horváth) (white-backed planthopper), and *Laodelphax striatellus* (Fallén) (small brown planthopper) (Hemiptera: Delphacidae), are the most economically important pests of rice^2–6^. They can cause serious yield losses both directly by feeding on phloem sap, causing the characteristic symptom ‘hopper burn’, and indirectly by transmitting viral diseases, resulting in billions of dollars of economic loss annually throughout Asia^2,3^.

Each of the three rice planthopper species can develop resistance to various insecticides independently^7–9^. Furthermore, in most situations, *N*. *lugens* is the most serious pest of rice, while the other two rice planthopper species are normally less serious pests than *N*. *lugens*^4,10^. Therefore, it is important to monitor the abundance of each of the three rice planthopper species in paddy fields for their management. Importantly, although two of the three rice planthopper species, *N*. *lugens* and *S*. *furcifera*, are unable to overwinter successfully in temperate areas of East Asia, including Japan, Korea, and most of China, re-colonization by these two species occurs annually in these areas following long-distance migration from overwintering areas, such as southern China and northern Vietnam^11^. *Laodelphax striatellus*, which can overwinter in temperate areas of East Asia, is also able to migrate overseas from eastern China to Japan and Korea^12^. Consequently, in these temperate areas, the abundance and composition of the three rice planthopper species in paddy fields are greatly influenced by the timing and magnitude of their migrations, which is not completely predictable^10,13^. Moreover, it is necessary to monitor the proportion of developmental stages in individuals of each rice planthopper species in paddy fields to decide when to apply insecticides^13^. In addition, adults of rice planthoppers show two wing-forms, macropterous (long-winged) form and brachypterous (short-winged) form, in both sexes. Macropterous adults tend to disperse in search of new habitats, whereas brachypterous adults cannot fly but have greater reproductive output than macropterous adults at the natal habitat^13,14^. Therefore, it is also important to monitor the proportion of adult wing-forms of each rice planthopper species in paddy fields for effective management practices. Thus, careful monitoring of the occurrence, reproduction, and population growth of the three rice planthopper species in paddy fields is necessary for their efficient management, especially in East Asia.

In surveys conducted to examine the abundance and composition of the three rice planthopper species in paddy fields, rice planthopper individuals are often captured on survey boards by the standard sticky board method^15^ (see Materials and Methods for details concerning this method). Traditionally, these surveys involve human visual inspection, wherein rice planthopper individuals captured on survey boards are sorted and counted by species, developmental stage, adult sex, and adult wing-form. This visual inspection task requires considerable time and effort by highly-trained experts. Therefore, the development of high-performance systems for automated classification and counting of rice planthopper individuals captured on survey boards has been a goal for a long time^13^. In recent years, deep learning has garnered considerable interest in many research fields, including computer vision. Notably, the development of methods based on convolutional neural networks (CNNs), which are deep learning techniques, has allowed computer vision to approach human performance on tasks such as detection and classification of objects in images^16^. We previously developed a deep learning-based object detection system which can automatically detect rice planthopper individuals from scanned images of survey boards and count the number of planthopper individuals according to 18 categories, including 17 categories based on a combination of species (*N*. *lugens*, *S*. *furcifera*, or *L*. *striatellus*), developmental stages (adult, late-instar [V instar] nymph, or mid-instar [III or IV instar] nymph), adult sexes (male or female), and adult wing-forms (macropterous form or brachypterous form), and one category, early-instar (I or II instar) nymph, with a mean average precision (mAP) of 79%^17^.

In this study, we improved the automated classification and counting system by reconsidering the categories of planthopper individuals to be counted and by additional supervised training. We also confirmed the time efficiency advantage of the automated classification and counting system by comparing the times required for automated classification and counting rice planthopper individuals with manual classification and counting.

## Results and discussion

### Categories of planthopper individuals for counting with the automated classification and counting system

The previous system counted the number of planthopper individuals captured on survey boards and sorted them into 18 categories (i.e., macropterous female [MF] of *N*. *lugens*, macropterous male [MM] of *N*. *lugens*, brachypterous female [BF] of *N*. *lugens*, brachypterous male [BM] of *N*. *lugens*, late-instar nymph [LN] of *N*. *lugens*, mid-instar nymph [MN] of *N*. *lugens*, MF of *S*. *furcifera*, MM of *S*. *furcifera*, BF of *S*. *furcifera*, LN of *S*. *furcifera*, MN of *S*. *furcifera*, MF of *L*. *striatellus*, MM of *L*. *striatellus*, BF of *L*. *striatellus*, BM of *L*. *striatellus*, LN of *L*. *striatellus*, MN of *L*. *striatellus*, and early-instar nymph). However, our previous study did not include a careful examination of planthopper species other than the three rice planthopper species through the automated system^17^. Furthermore, the categories of planthopper individuals which are rarely collected from paddy fields (i.e., less than 5% occurrence) could be excluded from those recognized and counted by the automated system. In practice, it is nearly impossible to obtain enough training and test datasets for these low-frequency categories. Previous studies indicate that brachypterous males of *S*. *furcifera*, which are sterile individuals due to the effects of parasites (e.g., *Agamermis unka* Kaburaki and Imamura [Nematoda: Mermithidae]), and brachypterous males of *L*. *striatellus*, which typically appear in overwintering individuals living outside of paddy fields, are very rare in paddy fields^13,18–21^, but our previous study excluded only brachypterous males of *S*. *furcifera* from the categories counted by the automated system^17^.

**Fig. 1:**
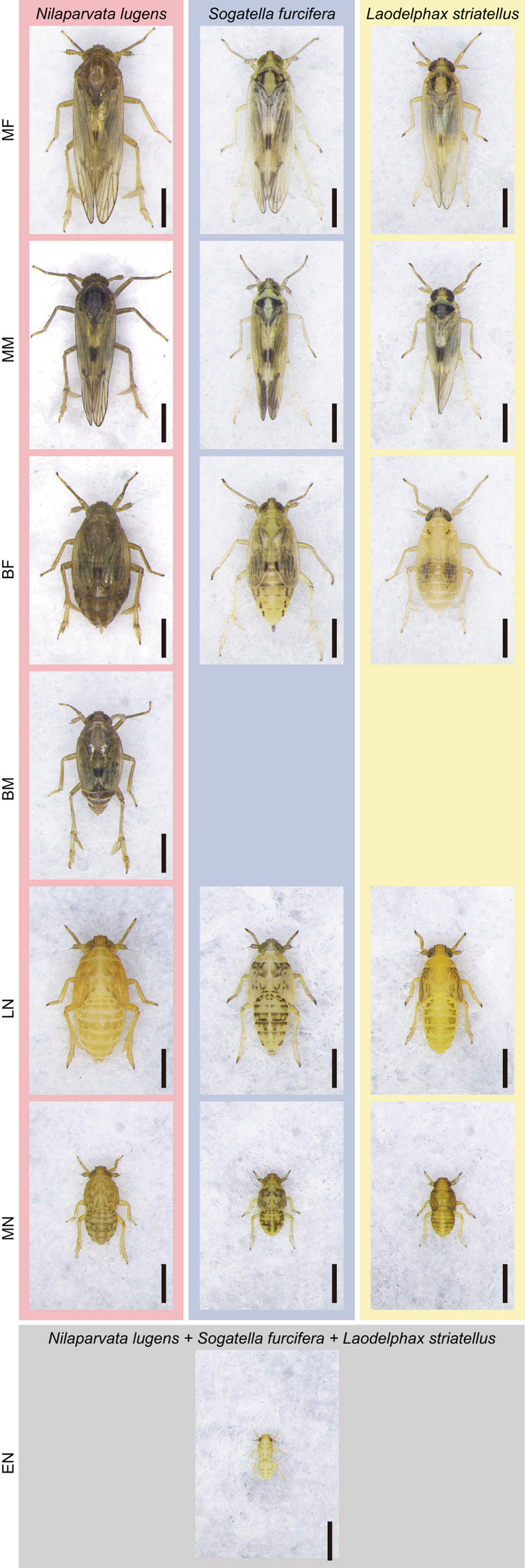
Seventeen categories of rice planthopper individuals for counting with the modified automated classification and counting system. Only individuals of early-instar nymphs could not be classified to the species level based on morphological characteristics. *MF* macropterous (long-winged) female, *MM* macropterous male, *BF* brachypterous (short-winged) female, *BM* brachypterous male, *LN* late-instar (V instar) nymph, *MN* mid-instar (III or IV instar) nymph, *EN* early-instar (I or II instar) nymph. Scale bars, 1 mm.

**Fig. 2:**
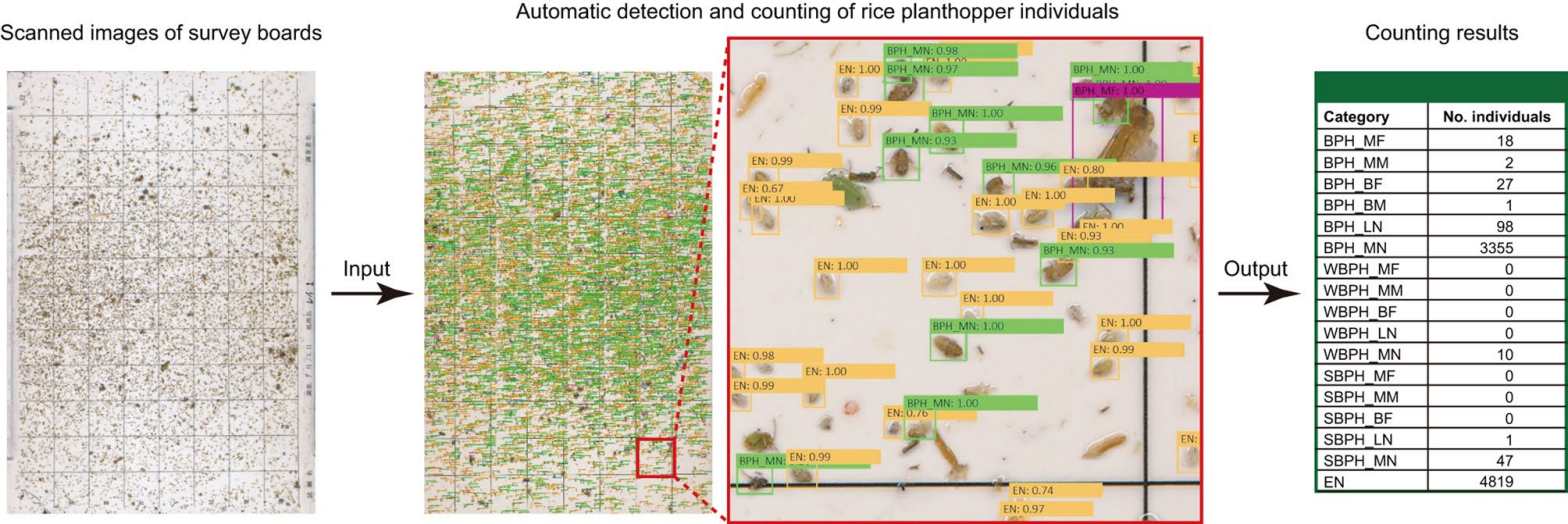
The modified automated rice planthopper classification and counting system. When scanned images of survey boards on which rice planthoppers have been captured are input into the system, rice planthopper individuals are automatically detected from the images and counted by 17 categories (see Fig. 1 for details concerning the 17 categories).

The planthopper categories that should be counted by the automated system were reconsidered with the dataset of planthopper individuals captured on survey boards in paddy fields at the Kyushu Okinawa Agricultural Research Center (Koshi, Kumamoto) and other paddy fields in western Japan (Supplementary Table S1) by the standard sticky board method^15^. A total of 192,385 planthopper individuals, comprising 18,394 adults (6652 macropterous females, 7044 macropterous males, 3400 brachypterous females, and 1298 brachypterous males), 22,993 late-instar nymphs, 44,047 mid-instar nymphs, and 106,951 early-instar nymphs, were captured on survey boards. Excluding early-instar nymphs, which cannot be identified accurately to the species level based on morphological characteristics^22,23^, each planthopper individuals could be morphologically identified to the species level, where all but seven adult individuals (i.e., more than 99.9% of adult individuals), and all but two individual nymphs (i.e., more than 99.9% of nymphal individuals) were identified as one of the three rice planthopper species. These results demonstrate that nearly all planthopper individuals captured on survey boards by the standard sticky board method belong to one of the three rice planthopper species. Moreover, eleven of 1823 individuals (i.e., only 0.6% of individuals) were brachypterous in male adults of *S*. *furcifera* and 47 of 1210 individuals (i.e., only 3.9% of individuals) were brachypterous in male adults of *L*. *striatellus* (Table 1), indicating that brachypterous males of both *S*. *furcifera* and *L*. *striatellus* are very rare in paddy fields, which is consistent with previous studies^13,18–21^. Therefore, we reached the conclusion that the automated classification and counting system does not need to count the number of brachypterous males of *S*. *furcifera*, those of *L*. *striatellus*, and individuals of planthopper species other than the three rice planthopper species to provide accurate and useful data. Thus, the system was modified by excluding the category of brachypterous males of *L*. *striatellus* (Fig. 1), and also by additional supervised training using scanned images of survey boards on which 63,510 individuals of the rice planthoppers had been captured (see “Materials and Methods” for details) (Fig. 2).

**Table 1:** Composition of planthopper individuals captured in paddy fields by the standard sticky board method.

Categories of planthoppers	No. individuals		
	Koshi, Kumamoto	Western Japan (except for Koshi, Kumamoto)	Total
*Nilaparvata lugens*			
Macropterous female (MF)	3260	58	3318
Macropterous male (MM)	3958	108	4066
Brachypterous female (BF)	2254	80	2334
Brachypterous male (BM)	1201	39	1240
Late-instar nymph (LN)	13,033	638	13,671
Mid-instar nymph (MN)	20,319	1381	21,700
*Sogatella furcifera*			
MF	1869	106	1975
MM	1696	116	1812
BF	130	58	188
BM	9	2	11
LN	4394	561	4955
MN	8250	2486	10,736
*Laodelphax striatellus*			
MF	1297	59	1356
MM	1106	57	1163
BF	829	48	877
BM	46	1	47
LN	4169	197	4366
MN	11,160	450	11,610
Other planthopper species	7	2	9
	(*Epeurysa nawaii* [1 MF], *Ishiharodelphax matsuyamensis* [1 BF], *Sogatella kolophon* [1 MF], *Sogatella vibix* [1 MM], *Stenocranus pacificus* [1 MF and 1 MM], *Unkanodes sapporonus* [1 MM])	(*Sogatella vibix* [1 LN and 1 MN])	
Early-instar nymph^a^	99,109	7842	106,951

^a^Individuals of early-instar nymphs could not be identified to the species level based on morphological characteristics.

### Evaluation of the modified automated planthopper classification and counting system

An overall assessment of the modified automated rice planthopper classification and counting system was evaluated with mAP, which is widely used for assessing the performance of object detection and classification tasks^24,25^. In the modified system, overall mAP at an intersection over union (IoU) threshold of 0.5 for all 17 categories reaches 91% with precision, recall, and F1-score of 84%, 93%, and 88%, respectively, where the average precision (AP) of each category ranges from 79 to 99% (Table 2). Remarkably, the mAP of all seven categories (i.e., MF, MM, BF, BM, LN, and MN of *N*. *lugens*, and early-instar nymph) concerning *N*. *lugens*, which is the most serious pest of rice in most situations^4,10^, averages 95% (Table 2). These results show that the modified system can detect, categorize, and count rice planthopper individuals in 17 categories with high precision.

**Table 2:** Performance of the improved automated classification and counting system.

Categories of rice planthoppers	Performance
*Nilaparvata lugens* (BPH)	
Macropterous female (MF)	AP = 94%
Macropterous male (MM)	AP = 99%
Brachypterous female (BF)	AP = 92%
Brachypterous male (BM)	AP = 89%
Late-instar nymph (LN)	AP = 97%
Mid-instar nymph (MN)	AP = 96%
*Sogatella furcifera* (WBPH)	
MF	AP = 98%
MM	AP = 93%
BF	AP = 79%
LN	AP = 96%
MN	AP = 86%
*Laodelphax striatellus* (SBPH)	
MF	AP = 93%
MM	AP = 84%
BF	AP = 85%
LN	AP = 92%
MN	AP = 81%
BPH, WBPH, or SBPH	
Early-instar nymph	AP = 96%
All 17 categories	mAP = 91% (Precision = 84%, Recall = 93%, F1-score = 88%)
All 7 categories concerning *N*. *lugens*	mAP = 95%

AP, average precision at an intersection over union (IoU) threshold of 0.5; mAP, mean average precision at an IoU threshold of 0.5.

The time efficiency of the automated classification and counting system was also evaluated by comparing the times required for classification and counting of rice planthopper individuals captured in paddy fields during planthopper outbreaks using the standard sticky board method^15^ by manual versus automated classification and counting. For two human experts on conventional visual inspection techniques for classification and counting of rice planthopper individuals, it took an average of 52.2 min (range = 24.9–71.1 min) per survey board to complete the visual inspection task (Table 3). On the other hand, by using the automated system, it took an average of 5.9 min (range = 5.3–6.5 min) per survey board to complete the classification and counting task (Table 3). A statistically significant difference was observed in the time required to complete the classification and counting task between the former and the latter (*t*_10_ = 5.99, *P* < 0.001; two-tailed *t*-test). These results show that the modified system greatly saves time and reduces labor costs for surveys to examine the abundance and composition of the three rice planthopper species in paddy fields.

**Table 3:** Time efficiency of the automated classification and counting system.

Survey board code^a^	Operators	Time required for the classification and counting task (min)	
		By the automated classification and counting system	By human experts
220929A	Anonymous researcher I	6.1	57.8
220929B	Anonymous researcher I	5.5	71.1
220929C	Anonymous researcher I	5.3	68.2
221011A	Anonymous researcher II	6.5	33.1
221011B	Anonymous researcher II	6.2	24.9
221011C	Anonymous researcher II	6.0	58.0
Mean (SEM)		5.9 (0.2)	52.2 (7.7)

^a^Numbers in survey board codes indicate the dates when surveys were conducted. For example, survey board 220929A was used in the survey on 29 September 2022.

## Conclusion

Although many species of insect pests, including rice planthoppers, have become able to be detected and classified automatically by deep learning-based object detection systems in recent years, automated classification of within-species categories of insect pest individuals by such systems is still challenging^26–29^. In this study, we developed a high-performance system for automated classification and counting of rice planthopper individuals captured on survey boards. This system can count rice planthopper individuals in 17 categories, which include within-species categories based on developmental stages, sexes, and wing-forms, with a mAP of 91% (Table 2) in about one-ninth of the time it takes a planthopper expert to provide the data manually (Table 3). The system developed here can greatly save time and reduce labor costs for monitoring the occurrence, reproduction, and population growth of the rice planthoppers in paddy fields, which could be useful for their efficient management directly as well as indirectly through better understanding of their biology.

When using the automated classification and counting system developed here, survey board images should be acquired by the scanners which are difficult to use in the field. Very recently, another method using images captured by augmented reality (AR) glasses, which can be used easily in the field, has been shown for the automated classification and counting of rice planthoppers on white plates. This system can count rice planthopper individuals on white plate across ten categories (i.e., macropterous adult [MA] of *N*. *lugens*, brachypterous adult [BA] of *N*. *lugens*, late and mid-instar nymph [LMN] of *N*. *lugens*, MA of *S*. *furcifera*, BA of *S*. *furcifera*, LMN of *S*. *furcifera*, MA of *L*. *striatellus*, BA of *L*. *striatellus*, LMN of *L*. *striatellus*, and early-instar nymph) with a mAP of 80%^30^. In addition to further challenges for improvement on the accuracy of automated classification and counting in each system (e.g., the application of the slicing-aided hyper inference technique^31^ to the system developed here), it would be important to use different automated planthopper classification and counting systems depending on the purpose of the survey.

## Materials and methods

### Composition of planthopper individuals captured in paddy fields using the standard sticky board method

To reconsider the categories of planthopper individuals to be counted by an automated classification and counting system, we investigated the composition of planthopper individuals captured on survey boards in paddy fields at the Kyushu Okinawa Agricultural Research Center (Koshi, Kumamoto) and other paddy fields in western Japan from 2020 to 2023 (Supplementary Table S1) by the standard sticky board method^15^. A rectangular plastic survey board (18 × 25 cm) coated with a sticky substance (Kinryu^®^ Spray; SDS Biotech, Tokyo, Japan) was held horizontally about 2 cm above the paddy water on one side of a rice hill at the bottom, and the rice hill was beaten twice by hand to cause planthoppers to drop onto the survey board. This procedure was repeated 20 times to collect planthoppers from 20 rice hills with one survey board. The planthoppers captured on the boards were transported to the laboratory, and individual planthoppers were counted by species, developmental stage, adult sex, and adult wing-form according to standard methods^20,23,32^. Early-instar nymphs were not identified to the species level because they cannot be identified accurately to the species level based on morphological characteristics alone^22,23,33^.

### Additional supervised training of the model for improved automated classification and counting

In our previous study, the model for the automated classification and counting system was trained on images of survey boards containing 15,559 individual rice planthoppers^17^. In this study, the model for the modified system was additionally trained on images of survey boards containing 63,510 rice planthoppers. All individuals were captured on survey boards in paddy fields at the Kyushu Okinawa Agricultural Research Center (Koshi, Kumamoto) from 2019 to 2022 using the standard sticky board method^15^. Scanned images of the survey boards were obtained using a CanoScan 9000F Mark II scanner (Canon Inc., Tokyo, Japan) or an EPSON GT-X830 scanner (Seiko Epson Corporation, Tokyo, Japan) at 1200 dpi. Each scanned image was divided into 96 images with a pixel resolution of 925 × 945. These images were manually annotated into 17 categories (i.e., MF of *N*. *lugens*, MM of *N*. *lugens*, BF of *N*. *lugens*, BM of *N*. *lugens*, LN of *N*. *lugens*, MN of *N*. *lugens*, MF of *S*. *furcifera*, MM of *S*. *furcifera*, BF of *S*. *furcifera*, LN of *S*. *furcifera*, MN of *S*. *furcifera*, MF of *L*. *striatellus*, MM of *L*. *striatellus*, BF of *L*. *striatellus*, LN of *L*. *striatellus*, MN of *L*. *striatellus*, and early-instar nymph) using LabelImg version 1.8.4 (https://github.com/tzutalin/labelImg). These 17 categories are independent of each other (i.e., no hierarchical rules were applied to the assignment of categories). The You Only Look Once (YOLO)-v7x algorithm was adopted as a detection model, and its program set was implemented with the deep neural network framework Darknet written in C language downloaded from github (https://github.com/AlexeyAB/darknet). This program set was compiled on Shiho, the supercomputer of NARO where the model was trained. The batch size was set to 65 with subdivisions of 13 as the training configuration. An original image of 925 × 945 pixels was resized to 960 × 960 prior to inputting to the model. The model was trained on 44,530 images which contained 63,510 planthopper individuals, and those images were randomly split into training and validation sets at a ratio of 8:2. Losses from the training and validation sets were calculated at each step (epoch). The model parameters were updated through the epochs so that the effect of losses on the training dataset decreased. The training process lasted for 51,000 epochs, and the model with the lowest validation loss was determined as the best one to be assessed for its final performance against the validation dataset.

### Evaluation of the performance of the modified automated classification and counting system

To evaluate the performance of the modified automated classification and counting system, we used test data images of survey boards containing a sum of 8250 individual rice planthoppers which had not previously been used as training data (Supplementary Table S2). Rice planthopper individuals were captured on survey boards in paddy fields in Kyushu, Japan from 2021 to 2023 (Supplementary Table S3) by the standard sticky board method^15^. Scanned images of the survey boards were obtained using an EPSON GT-X830 scanner (Seiko Epson Corporation, Tokyo, Japan) at 1200 dpi. Each scanned image was divided into 96 images with a pixel resolution of 925 × 945. In total, 5,664 images were obtained to test the trained model. These images were manually annotated into the 17 categories as mentioned above. This test dataset was used for assessing the trained model that had been determined as the best model in the training process. Each test image was resized from an original size of 925 × 945 to 960 × 960 pixels for input to the trained model, and detection results were obtained across all test images. This test process was done on the ‘Shiho’ supercomputer of NARO. The evaluation metrics were the AP for each category and mAP for the total categories at an IoU threshold of 0.5^24,25^. In addition, three other metrics that can be used to evaluate the model’s performance (i.e., Precision, Recall, and F1-score^34^) were calculated.

### Evaluation of the time efficiency of the automated classification and counting system

To evaluate the time efficiency of the automated classification and counting system, we compared the times required for classification and counting of rice planthopper individuals during rice planthopper outbreaks by human experts with the automated system. For the former, the time required to complete the visual inspection task per survey board for an expert was measured, where two anonymous researchers (who had already been trained for multiple years in conventional visual inspection techniques for classification and counting of rice planthopper individuals) from the Prefectural Agricultural Research Centers counted the number of rice planthoppers by species, developmental stage, adult sex, and adult wing-form. For the latter, the time required to complete the classification and counting task (which starts with the acquisition of a survey board image by the scanner, and ends with the automated classification and counting of rice planthopper individuals in the scanned image by the system) was measured, where the two anonymous researchers mentioned above used the system developed here with an EPSON GT-X830 scanner (Seiko Epson Corporation, Tokyo, Japan) and a NVIDIA Jetson AGX Xavier™ Developer Kit (https://www.sensefly.com/camera/parrot-sequoia/). To compare the times required for classification and counting of rice planthopper individuals between the former and the latter, we used a two-tailed *t*-test (JMP 13; SAS Institute, Cary, NC, USA).

## Electronic supplementary material

Below is the link to the electronic supplementary material.

Supplementary Material 1

## Data Availability

The datasets for the development and evaluation of the automated classification and counting system in the current study are available from the corresponding author on reasonable request. All other study data are included in the article and/or Supporting Information.
