# Supplementary material for: Development and evaluation of an automated classification and counting system for rice planthoppers captured on survey boards
Source: Sci Rep. 2025 Jul 1;15:22078. doi: 10.1038/s41598-025-05908-y (PMC12214890; doi:10.1038/s41598-025-05908-y)
Supplement: Supplementary file 1 — Supplementary Material 1 [file 41598_2025_5908_MOESM1_ESM.pdf]

Supplementary Information for

Development and evaluation of an automated classification and counting system for rice planthoppers captured on survey boards

Toshihisa Yashiro, Tomohiko Takayama, Ryo Sugiura, Masaya Matsumura, & Sachiyo Sanada-Morimura

Corresponding author: Toshihisa Yashiro
Email: yashiro.toshihisa704@naro.go.jp

This PDF file includes:

Tables S1 to S3

Table S1. Survey information detail for the investigation of the composition of planthopper individuals captured in paddy fields using the standard sticky board method used in this study.

**Location**	**Survey period**	**No. survey boards used**
Koshi, Kumamoto, Kyushu, Japan	1 June–5 October 2020	150
	8 June–26 October 2021	576
	30 May–24 October 2022	297
	5 June–28 August 2023	159
Tenri, Nara, Honshu, Japan	6 October 2023	4
Gose, Nara, Honshu, Japan	6 October 2023	4
Jinsekikogen, Hiroshima, Honshu, Japan	30 August 2023	4
Jinsekikogen, Hiroshima, Honshu, Japan	27 September 2023	4
Hofu, Yamaguchi, Honshu, Japan	22 September 2023	4
Shimonoseki, Yamaguchi, Honshu, Japan	22 September 2023	4
Iyo, Ehime, Shikoku, Japan	12 October 2023	4
Chikugo, Fukuoka, Kyushu, Japan	23 June–5 September 2022	20
Saga, Saga, Kyushu, Japan	22 July 2022	4
Isahaya, Nagasaki, Kyushu, Japan	9 September 2021	4
Unzen, Nagasaki, Kyushu, Japan	12 July–30 August 2022	16
	18 July–20 September 2023	8
Aso, Kumamoto, Kyushu, Japan	24 August–28 September 2023	8
Miyazaki, Miyazaki, Kyushu, Japan	20 July 2023	4
Minamisatsuma, Kagoshima, Kyushu, Japan	9 August 2022	4
	21 July 2023	4
Ishigaki, Okinawa, Ishigaki Island, Japan	13 April 2023	4

Table S2. Rice planthopper individuals in survey board images for evaluation of the performance of the modified automated classification and counting system.

Categories of planthoppers	No. individuals
Nilaparvata lugens	
Macropterous female (MF)	23
Macropterous male (MM)	50
Brachypterous female (BF)	75
Brachypterous male (BM)	33
Late-instar nymph (LN)	400
Mid-instar nymph (MN)	1523
Sogatella furcifera	
MF	68
MM	70
BF	55
LN	205
MN	422
Laodelphax striatellus	
MF	34
MM	28
BF	29
LN	101
MN	295
Early-instar nymph[a]	4839

[a]Individuals of early-instar nymphs cannot be identified to the species level based on morphological characteristics.

Table S3. Survey details for the evaluation of the modified automated classification and counting system in this study.

Location	Survey period	No. survey boards used
Chikugo, Fukuoka, Kyushu, Japan	23 June–5 September 2022	20
Saga, Saga, Kyushu, Japan	22 July 2022	4
Isahaya, Nagasaki, Kyushu, Japan	9 September 2021	4
Unzen, Nagasaki, Kyushu, Japan	12 July–30 August 2022	16
Koshi, Kumamoto, Kyushu, Japan	4 October 2021	4
Koshi, Kumamoto, Kyushu, Japan	16 August 2022	4
Koshi, Kumamoto, Kyushu, Japan	26 June 2023	3
Minamisatsuma, Kagoshima, Kyushu, Japan	9 August 2022	4
